# Production of α-cuprenene in *Xanthophyllomyces dendrorhous*: a step closer to a potent terpene biofactory

**DOI:** 10.1186/1475-2859-12-13

**Published:** 2013-02-05

**Authors:** Elena Melillo, Rita Setroikromo, Wim J Quax, Oliver Kayser

**Affiliations:** 1 Department of Pharmaceutical Biology, Groningen Research Institute of Pharmacy, University of Groningen, A. Deusinglaan 1, Groningen 9713AV, The Netherlands; 2 Department of Technical Biochemistry, Technical University of Dortmund, Emil-Figge-Strasse 66, Dortmund 44227, Germany

**Keywords:** *Xanthophyllomyces dendrorhous*, α-cuprenene, Metabolic engineering, *Escherichia coli*, *Saccharomyces cerevisiae*, Terpene cell factory

## Abstract

**Background:**

The red yeast *Xanthophyllomyces dendrorhous* is a natural producer of the carotenoid astaxanthin. Because of its high flux, the native terpene pathway leading to the production of the tetraterpene is of particular interest as it can be redirected toward the production of other terpene compounds. The genetic tools for the transformation of the yeast with the concurrent knock-out of genes involved in the astaxanthin biosynthesis are made available and here we show that the production of the sesquiterpene α-cuprenene is possible in mutant strains of *X. dendrorhous* transformed with the *Cop6* gene originating from the fungus *Coprinus cinereus*. For the evaluation of the production levels, we chose to express the same gene and analyze the accumulation of α-cuprenene in *Escherichia coli* and *Saccharomyces cerevisiae*, as well. Here we propose that *X. dendrorhous* is a candidate in the search for the potential platform organism for the production of terpenes.

**Results:**

All three *X. dendrorhous* mutants functionally express the *Cop6* gene and accumulate α-cuprenene. The production of α-cuprenene in the red yeast reached 80 mg/L, which represents a far higher concentration compared to the levels obtained in the *E. coli* and *S. cerevisiae* mutants. At this expression levels the pool of terpene precursors has not become a limiting factor in the *X. dendrorhous* mutants since the expression of the *Cop6* gene in the genomic rDNA of the yeast allows production of both α-cuprenene and astaxanthin without affecting the growth or the accumulation levels of both compounds.

**Conclusions:**

We have shown that *X. dendrorhous* can produce α-cuprenene, and the results here presented, next to the capability of accumulating at least two more non-native sesquiterpenes, demonstrates the high potential of this yeast to become an interesting terpene-based drugs producer.

## Background

Since ancient times, microorganisms have been used to produce bread, wine and dairy products in order to improve the quality of food and its nutrients. Today, microbes are utilized for the manufacture of a wide variety of fine or bulk chemicals including antibiotics [1,2], vitamins [3], biofuels [4,5], biodegradable and biocompatible plastics from waste [6] and terpene-based drugs [7,8]. Originally, the choice for a production host was dictated by the ability of the organism to produce the desired compounds, but in most cases the concentration of the chemicals of interest was not sufficient to cover the market demand.

With the development of genetic engineering, new tools became available to overcome these obstacles. The possibility of transferring single genes or even complete pathways to other microorganisms led to improved yields or easier bioprocessing conditions. Furthermore, metabolic engineering allowed the enhancement of the yields in the native hosts by finely tuning the metabolic networks of the cells towards the optimized production of the specific compound. More recently, the combination of synthetic biology and metabolic engineering has resulted in the creation of hosts capable of producing a non-native compound with high efficiency obtainable only by optimizing the native pathways of the cells [9].

The majority of the yeast or bacterial strains used by industry have been selected for the several advantages they deliver: they are easy to cultivate, they can grow on cheap media, they are generally regarded as safe (GRAS status) and their metabolic pathways are easy to modify via genetic engineering.

*Xanthophyllomyces dendrorhous*, a red basidiomycetous yeast, represents one of the microbial strains already used in industry and shares all the aforementioned advantages [10]. Today, *X. dendrorhous* is grown at industrial scale for its native capability to produce the valuable carotenoid astaxanthin.

Carotenoids, together with several other pharmaceutically important compounds, like artemisinin and Taxol, belong to the natural compounds class of the terpenes [11]. Several efforts have been put in the engineering of a platform organism for the production of industrially important terpenes [7,8].

We hypothesize that, since *X. dendrorhous* can produce high levels of astaxanthin, which shares the same precursors with all other terpenes, it can also utilize those same precursors for the production of any other terpenoid compound.

The red yeast was already shown to be able to functionally express the pentalenene synthase from a *Streptomyces* strain involved in the biosynthesis of the antibiotic pentalenolactone [12]. In order to further evaluate the potential of the red yeast as a platform organism for terpenes, we expressed the sesquiterpene cyclase *Cop6* in *X. dendrorhous* mutant strains. The protein Cop6, originating from the fungus *Coprinus cinereus*, produces the cyclized sesquiterpene α-cuprenene, which is the basic structure for the formation of lagopodin A, an antimicrobial sesquiterpene quinone [13]. We also compared the accumulation levels of α-cuprenene with two of the most industrially utilized microbial strains, *E. coli* and *S. cerevisiae*.

## Results

### Production of cuprenene in *E. coli*

Isolation of volatile terpenoids in *E. coli* via addition of a dodecane organic phase to the liquid cultures has been shown to be extremely efficient [14]. We have decided to adopt the same strategy to capture the α-cuprenene produced by the *E. coli* strains transformed with the *Cop6* gene. In order to be able to compare the levels of α-cuprenene at the different time points and from different organisms we added hexadecane in known concentrations, as an internal standard, to the dodecane.

**Figure 1:**
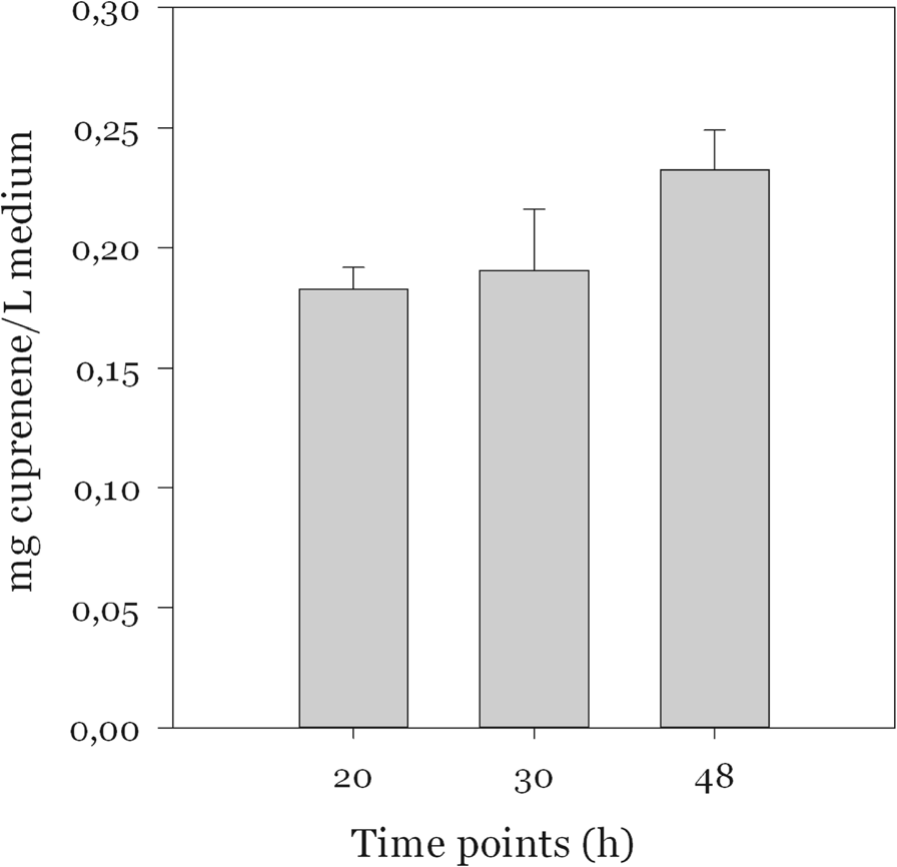
**Cuprenene production during time course with *E. coli *pHis8Cop6.**

The wild type strain and the transformed one exhibited the same growth curve and biomass accumulation; twenty hours after induction of the expression, the cells reached the highest density and then started dying after 30 hours. After 48 hours the concentration of cuprenene, based on the internal standard, in the modified strain reached approximately 0.25 mg/L of culture, a slight increase compared to the concentration at 20 hours (Figure 1).

### Growth curves and α-cuprenene production from *S. cerevisiae* and *X. dendrorhous* in rich medium

**Figure 2:**
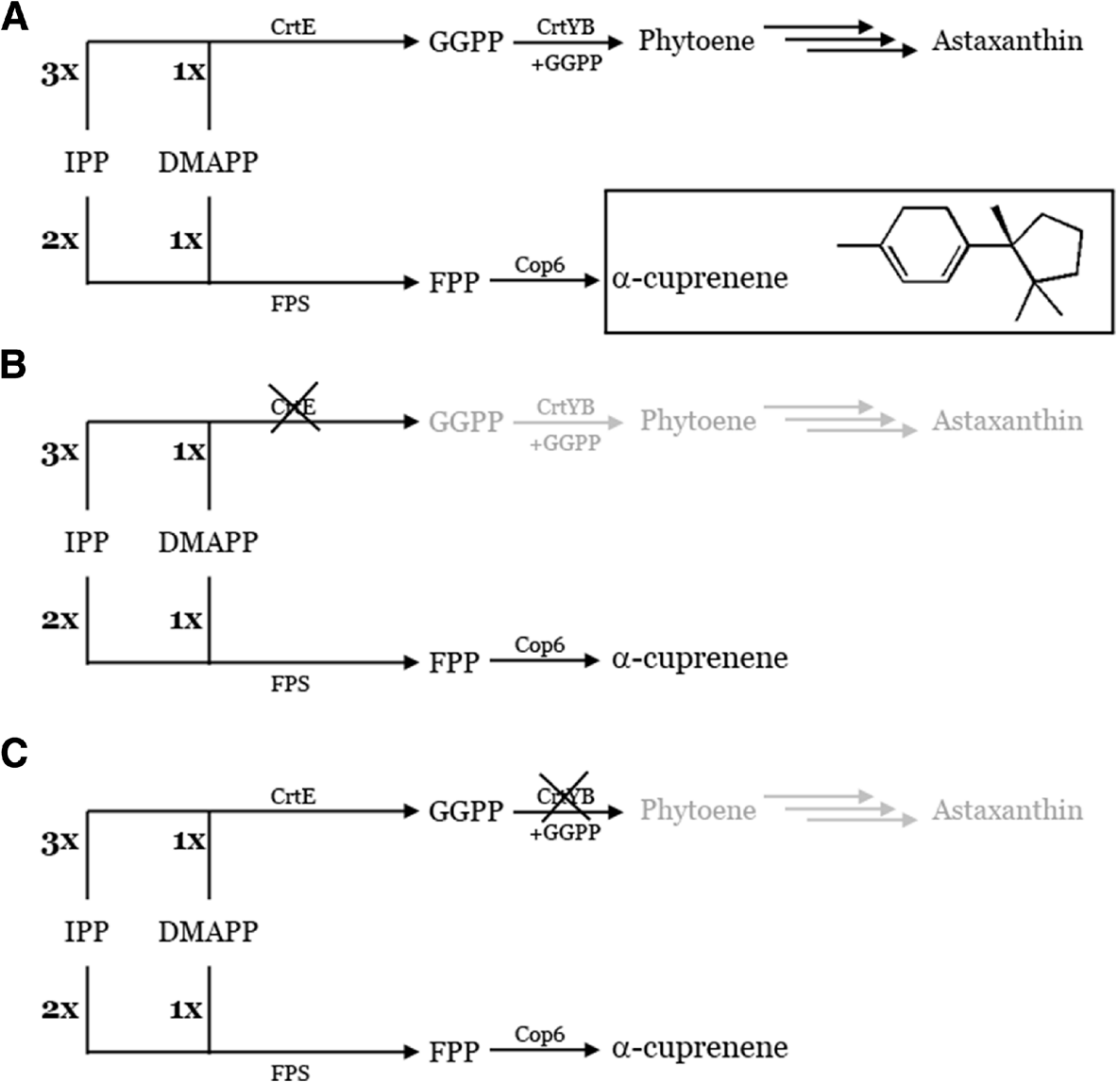
**Schematic representation of *X. dendrorhous *mutant strains.** (**A**) In the mutant *XdCop6*, the native astaxanthin pathway has not been modified but the gene *Cop6* has been integrated in the rDNA of the yeast allowing the mutant to produce both astaxanthin and α-cuprenene. (**B**) In the strain Δ*E-Cop6* the *Cop6* gene has been inserted in the *crtE* gene causing the disruption of the carotenoid production at the GGPP synthesis level. (**C**) When *Cop6* is inserted in the *crtYB* gene, the Δ*YB-Cop6* strain is created. While there is still expression of the GGPPS, phytoene cannot be produced anymore, blocking the production of astaxanthin one step downstream of the Δ*E-Cop6*.

After the separate transformations of *X. dendrorhous* wild-type strain with the vectors pCrtE-Cop6, pCrtYB-Cop6 and pPR-Cop6, one colony from each transformation plate was chosen to be grown and analyzed. As expected, since the astaxanthin pathway was disrupted (Figure 2), on the plates used to select *ΔE-Cop6* and *ΔYB-Cop6*, the mutant colonies presented a white phenotype. In contrast, *XdCop6* colonies, transformed with pPR-Cop6, in which the carotenoid pathway was not modified (Figure 2), shared an orange pigmentation with the wild type strain.

The *S. cerevisiae* mutant, *ScCop6*, was isolated after transformation of the wild type strain of *S. cerevisiae* with the plasmid p426GPD-Cop6, which allows constitutive expression of the *Cop6* gene.

A time course analysis was performed on the *ScCop6* and on the three *X. dendrorhous* mutant strains, *XdCop6*, *ΔE-Cop6* and *ΔYB-Cop6*. The strains were grown in YPD medium in order to obtain a high growth in shake flasks. The time course consisted of four sampling times after the inoculation in fresh medium; we chose 24 h, 48 h, 72 h and 96 h to be able to observe all the steps of the growth curve.

With respect to the OD_600_ and to the cell dry weight, the *S. cerevisiae* and *X. dendrorhous* mutants exhibit similar curves compared to the respective wild type strains.

**Figure 3:**
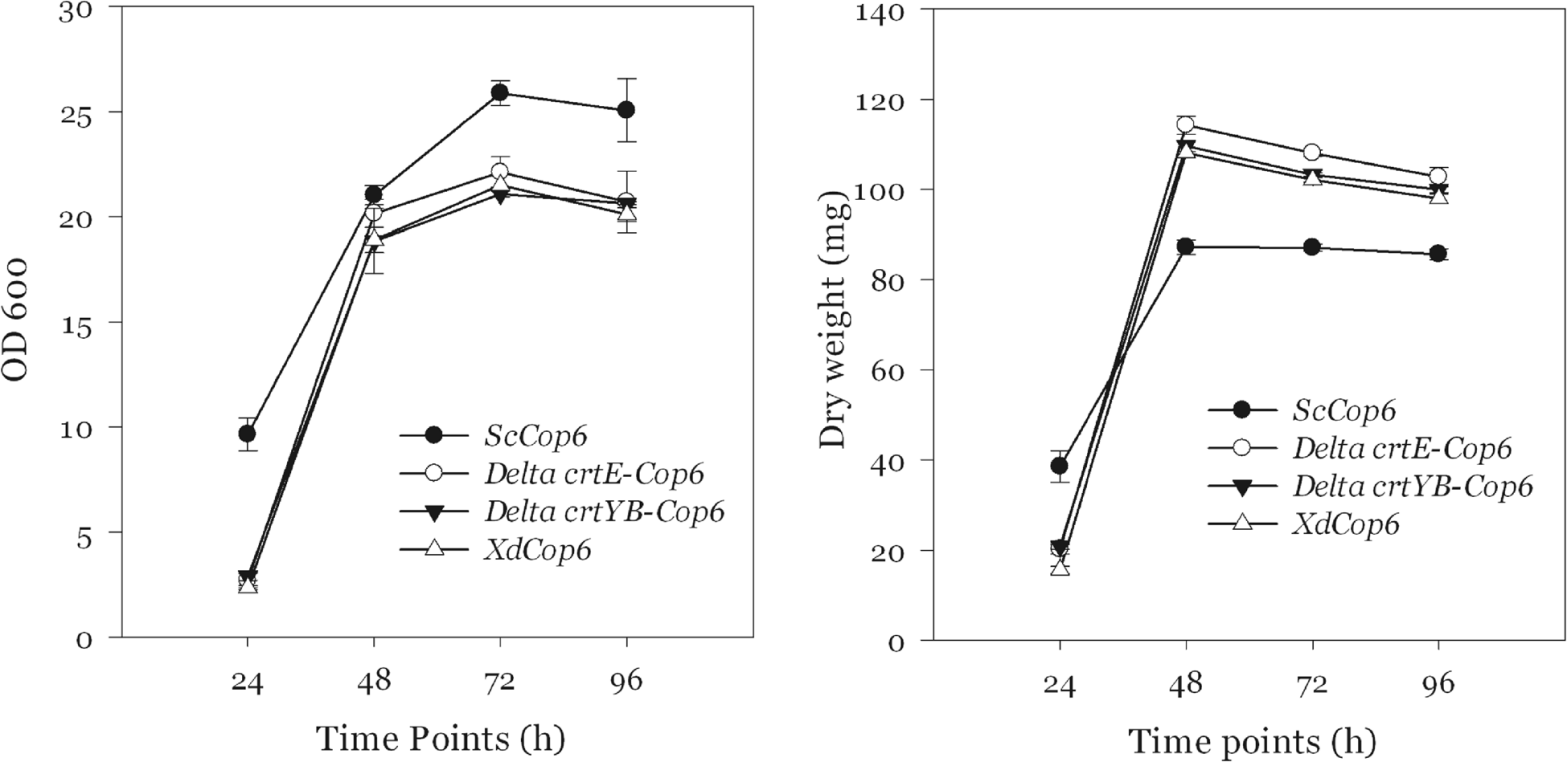
**OD**_**600 **_**and cell dry weight of *ScCop6*, *ΔE-Cop6*, *ΔYB-Cop6 *and *XdCop6 *in 10 ml of YPD medium.**

Figure 3 compares the growth curves obtained during the time course analysis from the mutant strains of both yeasts. In spite of the fact that *S. cerevisiae* cells reach a higher optical density, they accumulate a lower biomass compared to all *X. dendrorhous* strains.

**Figure 4:**
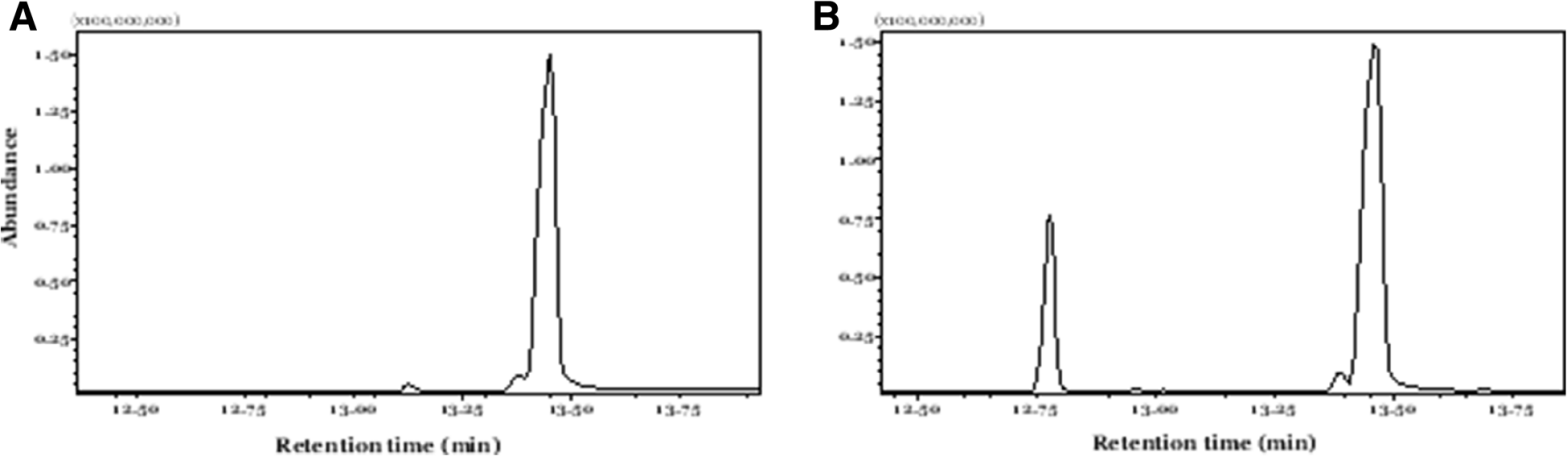
**Chromatograms of the diluted dodecane from (A) *X. dendrorhous *wild type strain and (B) *ΔE-Cop6*.** The peak at 13.5 minutes is the hexadecane used as internal standard. The α-cuprenene has a retention time of approximately 12.8 minutes.

**Figure 5:**
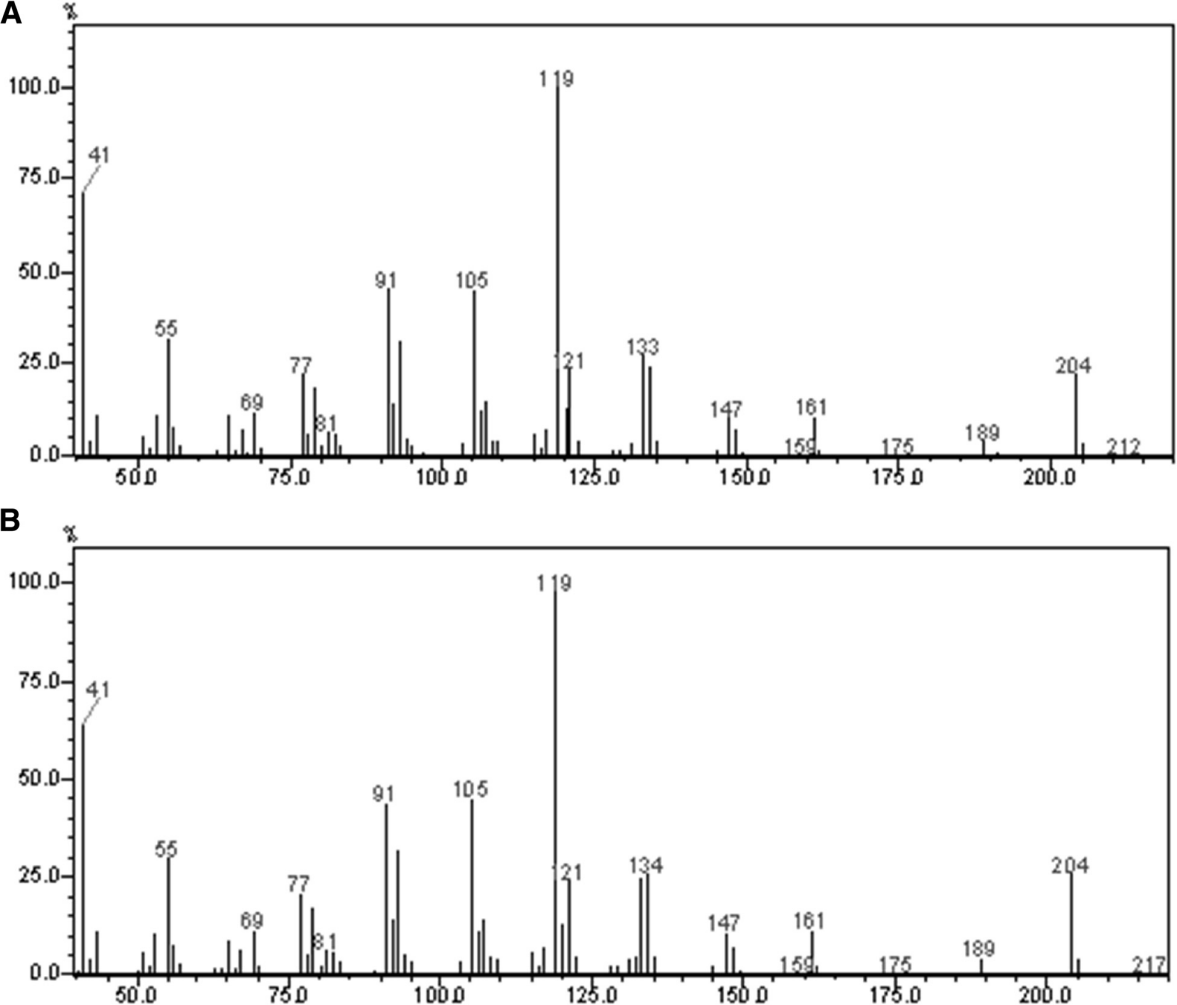
**Fragmentation patterns.** (**A**) α-cuprenene from *ScCop6*; (**B**) peak at 12.8 minutes from *XdCop6*. The “x” axis represents the m/z ratio.

The diluted dodecane solutions from the *S. cerevisiae* strains and from the wild type and mutant *X. dendrorhous* were analyzed by GCMS, and a single peak appeared in the chromatograms from the mutants at 12.8 minutes (Figure 4). The fragmentation pattern of the peak was compared to the pattern corresponding to α-cuprenene produced in *S. cerevisiae* (Figure 5). The mass and the relative ratio of the fragment peaks matched between the two patterns, allowing us to confirm that the only sesquiterpene produced by Cop6 in *X. dendrorhous* is α-cuprenene.

**Figure 6:**
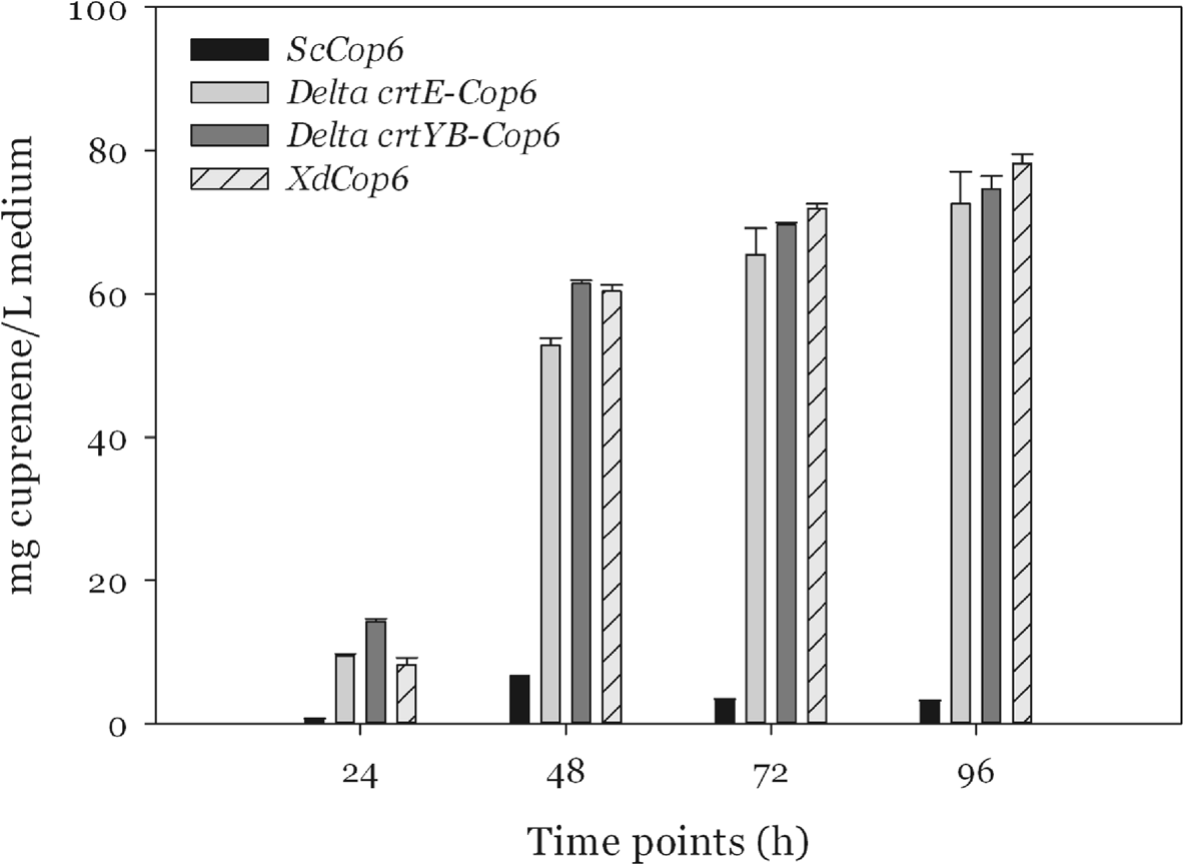
Production of α-cuprenene in rich YPD medium.

The time-course production of α-cuprenene in the four strains is represented in Figure 6. The level of the sesquiterpene in *S. cerevisiae* sharply increased after 24 hours to reach a maximum of 6.6 mg/ L on the second day of culturing and then appeared to decrease during the following two days.

The α-cuprenene production in the three *X. dendrorhous* strains showed ten times higher levels with a major increase between 24 and 48 hours, as well. In contrast to *S. cerevisiae*, *XdCop6*, *ΔE-Cop6* and *ΔYB-Cop6* constantly produced α-cuprenene during the complete time course. The accumulation of the sesquiterpene appeared to be consistent in all the three *X. dendrorhous* strains and was directly proportional to the cell mass, in particular in the first three days after inoculation. The highest yield was obtained after 96 hours with the *XdCop6* strain and corresponded to almost 80 mg/L of culturing medium. *ΔE-Cop6* could produce up to 70 mg of α-cuprenene per liter of medium and *ΔYB-Cop6* 74 mg/L, both after growing for 96 hours.

### Growth curves and α-cuprenene production from *S. cerevisiae* and *X. dendrorhous* in minimal medium

The *ScCop6* mutant was selected on medium lacking uracil in order to allow only the colonies containing the plasmid to grow. The rich YPD medium contains all the necessary nucleotides, thus the selective pressure on the *S. cerevisiae* mutant strain grown in this medium was inexistent. For this reason, we decided to grow all the strains in a minimal medium lacking uracil which would allow a more accurate comparison between the *ScCop6* and the three *X. dendrorhous* mutants. The same settings chosen for the time course in rich medium were applied for the growth and α-cuprenene production analysis in minimal medium.

An overall lower cell mass and OD_600_ compared to rich medium was observed for all the strains and no difference in growth could be discerned between the wild type strains and the mutant strains, both for *S. cerevisiae* and *X. dendrorhous*.

**Figure 7:**
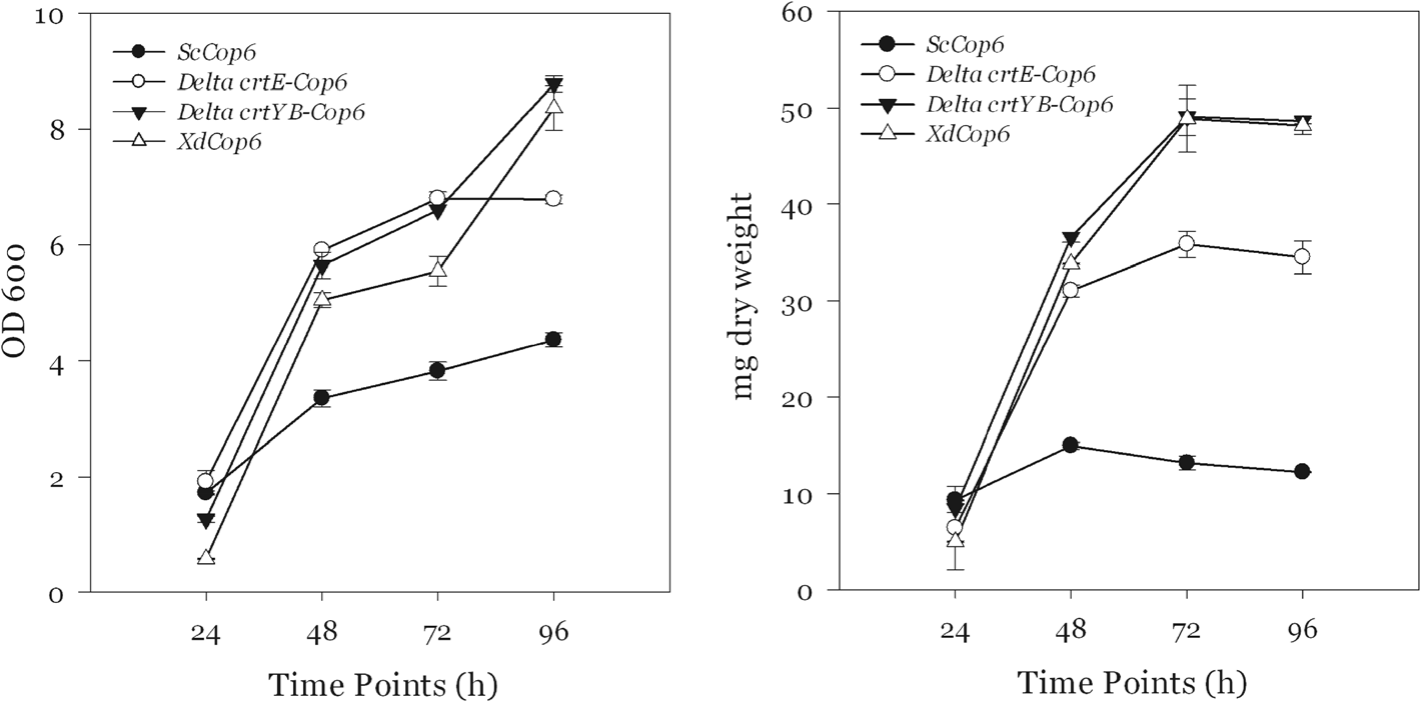
**OD**_**600 **_**and cell dry weight of *ScCop6*, *ΔE-Cop6*, *ΔYB-Cop6 *and *XdCop6 *in 10 ml of minimal medium.**

*S. cerevisiae* strains started growing already after 24 hours while *XdCop6*, *ΔE-Cop6* and *ΔYB-Cop6* showed a lag phase between 0 and 24 hours and a log growth between 24 and 48 hours (Figure 7). In minimal medium *ΔE-Cop6* exhibited a slightly reduced cell growth compared to *XdCop6* and *ΔYB-Cop6.*

**Figure 8:**
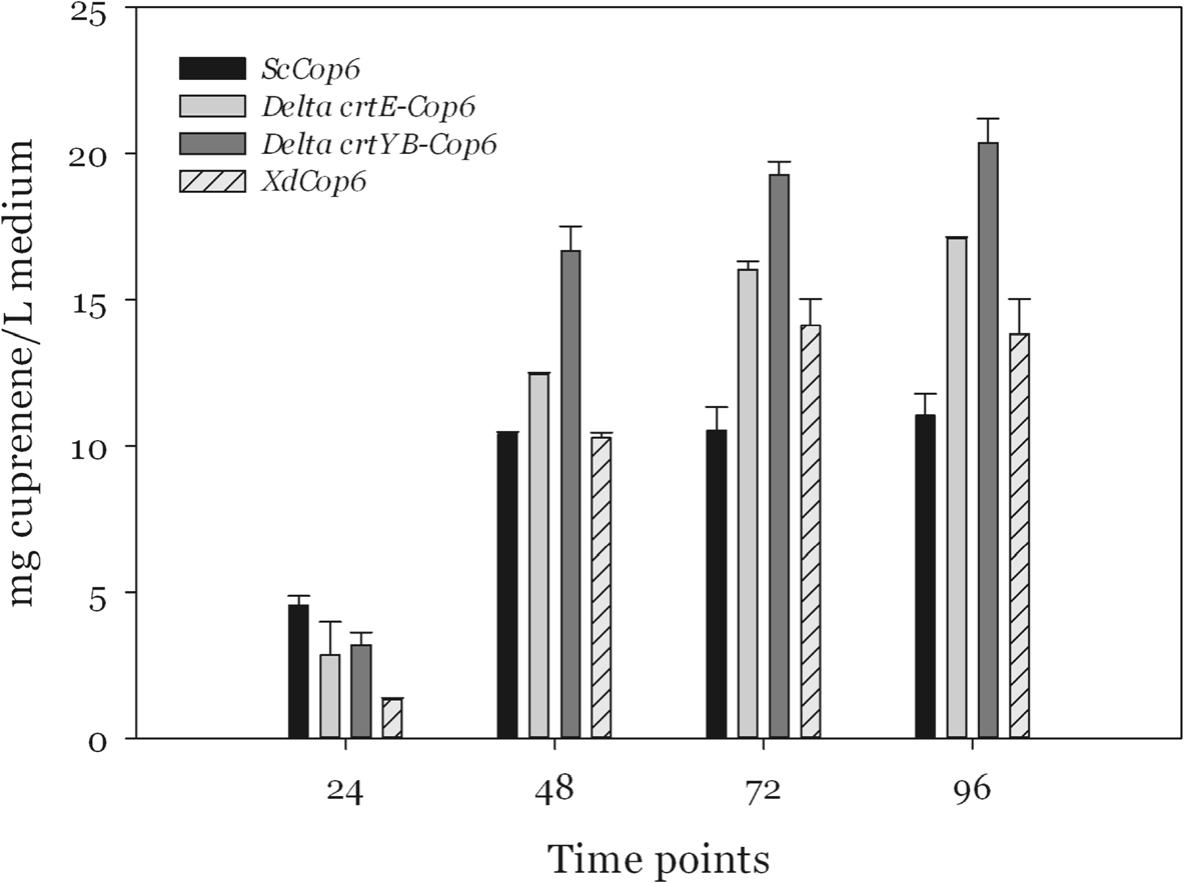
Production of α-cuprenene in minimal medium.

The accumulation of α-cuprenene in the three *X. dendrorhous* strains was significantly lower than the concentration obtained in YPD reaching a maximum of 20 mg/l (Figure 8) for *ΔYB-Cop6*. When *ScCop6* was grown in minimal selective medium, the sesquiterpene production increased nearly to 12 mg of α-cuprenene per liter of medium.

### Microscopy analysis of *ScCop6* and *XdCop6*

Figures 3 and 7 indicate that *S. cerevisiae* and *X. dendrorhous* are characterized by a different growth rate and optical density. For a better understanding of the differences between the two yeasts, we performed a microscopy analysis on the strains *ScCop6* and *XdCop6*.

A Burke chamber was used to count the yeast cells. In spite of the macroscopic red pigmentation of *XdCop6*, the cells appeared white under the microscope white light. After counting samples in duplo from *ScCop6* and *XdCop6* and calculating the cell numbers, we observed that one OD_600_ unit in *S. cerevisiae* corresponded to 3 x 10^7^ cells per milliliter, consistent with the data available in literature [15]. In contrast, the number of *X. dendrorhous* cells counted in one milliliter of culture with the same optical density was 4.8 x 10^6^.

**Figure 9:**
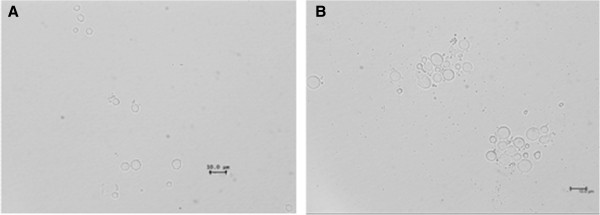
**Microscopy photographs.** (**A**) *ScCop6* and (**B**) *XdCop6*.

To further ascertain the morphological differences between *ScCop6* and *XdCop6*, we compared the average cells sizes. Figure 9 compares the pictures of the two strains after four days of growth; the same magnification was used to visualize the cells. Cells from *XdCop6* are round shaped and have a granular appearance, while *ScCop6* cells, although showing a similar round morphology, show a more homogeneous cytosol. On average, the size of *X. dendrorhous* cells was 10 μm in diameter; *S. cerevisiae* cells were smaller with a size ranging between 7 and 8 μm. Not only *ScCop6* cells were on average smaller than *X. dendrorhous*, but they also never had a diameter bigger than 10 μm, in contrast with the red yeast cells.

## Discussion

*E. coli* and *S. cerevisiae* are two model organisms that also serve as industrial cell factories for the production of a wide variety of compounds ranging from pharmaceutically active substances to food ingredients and biofuels.

In spite of the fact that *X. dendrorhous* has not been studied as extensively as *E. coli* or *S. cerevisiae*, it shows great potential to become a platform organism for terpene production [12]. In order to assess the value of the red yeast as a cell factory, we expressed the *Cop6* gene in the three *X. dendrorhous* mutants and compared the production of α-cuprenene with *E. coli* and *S. cerevisiae* strains expressing the same gene.

The cDNA from the *Cop6* gene was expressed by all *X. dendrorhous* mutants, whereas the genomic version of the gene, when transferred to the red yeast, did not result in α-cuprenene accumulation (data not shown) indicating that *X. dendrorhous* cannot correctly splice the gene from *C. cinereus*.

When grown in rich medium, all *X. dendrorhous* strains, including the wild type, showed very similar growth rates: they reached OD_600_ values of 20 and produced a maximum of 12 grams of cell dry weight per liter of culture. Similarly, when looking at the α-cuprenene production, the three strains *XdCop6*, *ΔE-Cop6* and *ΔYB-Cop6* did not show big differences among each other, with levels of the sesquiterpene ranging from 70 to 80 mg of compound per liter of medium.

Comparing Figures 3 and 7, it is clear that, differently from the experiments in the rich medium, the three *X. dendrorhous* strains shared an altered growth behavior in the minimal medium. While *XdCop6* and *ΔYB-Cop6* reached a maximum cell dry mass of nearly 5 g/L, similar to the one obtained with the wild type strain, *ΔE-Cop6* could not produce more than 3.5 g of dry cells per liter of medium. In 2008 Niklitschek and colleagues have reported the difficulty to isolate a *X. dendrorhous* strain in which both *crtE* alleles had been knocked out [16], suggesting an important role of this protein in the yeast growth. In the light of the results shown in this study, we can conclude that difference in growth between the *ΔE-Cop6* mutant and all the other *X. dendrorhous* strains in the minimal medium could be explained by a lack, in this particular medium, of compounds important for the yeast growth produced directly or indirectly by the CrtE protein.

Concomitantly with the reduced growth in the minimal medium, the concentration of α-cuprenene in the dodecane was also affected, reaching values ranging from 15 to 21 mg/L of culture. The decrease in sesquiterpene accumulation can partly be explained by the reduced cell mass and partly by the lower concentration of nutrients in the minimal medium which would induce the cells to minimize the energy consumption by shutting down unnecessary pathways.

When comparing cell mass accumulation and α-cuprenene production in all *X. dendrorhous*, *E. coli* and *S. cerevisiae* strains, the prokaryote showed the lowest values. The low biomass in the bacterium is most likely to be ascribed to a lack of glucose in its growth medium, while the limited sesquiterpene production is due to the lower terpene flux in *E. coli* compared to the two eukaryotes.

The differences in growth curves and dry weight between the *X. dendrorhous* and the *S. cerevisiae* strains seem to have morphological reasons. *X. dendrorhous* cells are on average bigger than *S. cerevisiae* ones [17] and at the same optical density *S. cerevisiae* cell counts are almost 10 times higher than in *X. dendrorhous* cultures, meaning that the same OD_600_ value corresponds to more *S. cerevisiae* cells than it does for *X. dendrorhous*. Since, at the beginning of the time course experiments, the initial OD_600_ for all the strains was set at 0.05, the number of cells initially transferred to the fresh medium was higher in *ScCop6* than in all the *X. dendrorhous* mutants. This would explain the delay in growth we observed for *XdCop6*, *ΔE-Cop6* and *ΔYB-Cop6* compared to *ScCop6*. Additionally, the higher cell mass accumulation observed in the *X. dendrorhous* strains compared to *S. cerevisiae* may be due to the red yeast’s bigger sized cells rather than to a higher number of cells.

The highest α-cuprenene production levels were obtained with the *X. dendrorhous* strains both in the rich and in the minimal medium experiments. Remarkably, in the YPD medium the gap in sesquiterpene accumulation between the red yeast and the *S. cerevisiae* strain was far more pronounced. We assume that, since the complete medium does not allow selective pressure on *ScCop6*, which was isolated by its ability to grow on minimal medium lacking uracil, the strain might have undergone a reduction in plasmid copy number.

While *ScCop6* mutants contain an average of 20 copies of *Cop6*, the *X. dendrorhous* white mutants possess just one copy of the gene since the recombination of the constructs can occur only once in the single *crtE* or *crtYB* genes. In order to obtain a mutant with a higher number of integrations of the gene in the genomic rDNA, we transformed the *X. dendrorhous* wild type strain with a higher concentration of the DNA fragment from the pPR-Cop6 vector and selected the transformants on YPD medium containing a concentration of geneticin 5 times higher than normal, hoping for gene amplification. Unfortunately, no colony grew after this transformation and we could not evaluate the effect of more gene copies on the α-cuprenene accumulation.

Nevertheless, we can safely assume that the concentration of the precursors is not a limiting factor in the sesquiterpene production in *X. dendrorhous*, since the strain *XdCop6* can easily sustain the production of both α-cuprenene and astaxanthin, especially when grown in the YPD rich medium. The production of both terpene compounds in *XdCop6* confirms the hypothesis that a higher gene copy number would positively influence the α-cuprenene production in *X. dendrorhous*.

In conclusion, *X. dendrorhous* shows great promise since it has the GRAS status, it grows at room temperature in minimal media, and it has already been used by industry for the production of astaxanthin. We discovered that it can produce at least three non-native sesquiterpenes, pentalenene [12], α-cuprenene and cubebol (data not shown). Furthermore, *X. dendrorhous* is the best microorganism, among the ones we have analyzed, to be used for the production of α-cuprenene. A better understanding of the molecular biology of this yeast will prove useful for the identification of stronger promoters for a higher gene expression.

In light of the aforementioned advantages and of the provided results, *X. dendrorhous* is an interesting candidate for being used as a cell factory for the production of terpenes.

## Methods

### Strains and culture conditions

The *E. coli* strain DH5α was used for the cloning processes, while the strain BL21 (DE3) was transformed and cultured for the time course experiments. *S. cerevisiae* MRG 5 #502 (*MATa*, *ura*3-52, *leu*2-Δ1, *trp*1-Δ36, *his*3-Δ200, D*ade*2) was used for the transformation and time course analysis. *X. dendrorhous* wild type strain (CBS 6938) was used for the transformations and as negative control for all experiments. Both *E. coli* strains were grown in LB (10 g/L Trypton, 5 g/L Yeast Extract, 10 g/L NaCl) with 30 mg/ml kanamycin. The rich medium for *S. cerevisiae* and *X. dendrorhous* was YPD (10 g/L Yeast Extract, 20 g/L Peptone and 20 g/L Dextrose) with additional 40 mg/ml geneticin (G-418 Sulphate, Gibco) only for the selection and growth of the *X. dendrorhous* mutants. The minimal medium for *S. cerevisiae* consisted of 13.4 g/l Yeast Nitrogen Base without amino acids, 20 g/L Dextrose, 100 mg/L leucine, 40 mg/L histidine, 40 mg/L tryptophan and 40 mg/L of uracil. The same concentrations of Yeast Nitrogen Base and Dextrose were kept for the minimal medium for *X. dendrorhous* and geneticin was added to the medium for the culturing of the mutants.

### Construction of the *E. coli* strain *EcCop6* and time course analysis

The plasmid pHis8Cop6 was a kind gift of Prof. Claudia Schmidt-Dannert from University of Minnesota and it contains the cDNA sequence of the *Cop6* gene from *Coprinus cinereus* under the control of the T7 promoter. *E. coli* BL21 (DE3) colonies containing pHis8Cop6 were selected on LB plates with kanamycin.

One transformed colony, *EcCop6*, was chosen for the time course analysis and a seed culture was started over night in LB plus kanamycin. The fresh cultures were inoculated and grown to OD_600_ 0.5 and then 1 mM isopropyl β-D-1-thiogalactopyranoside (IPTG) was added for the induction of expression of the *Cop6* gene. The time course was performed in duplo and consisted of six 100-ml flasks with 10 ml of LB medium plus kanamycin and 500 μl of dodecane mixed with the internal standard, hexadecane (68 μg/ml of medium), grown in a shaking incubator at 250 rpm at 37°C for 48 hours. Two flasks were removed from the incubator at each sampling point (20, 30 and 48 hours after IPTG induction); the cultures were centrifuged at 4000 rpm for 10 minutes, the upper dodecane layer was isolated from the medium and used for the GCMS analysis. The cell pellet was washed once with water and freeze dried to determine the cell dry weight. The same procedure was applied to *S. cerevisiae* and *X. dendrorhous*, as well.

### Construction of the *S. cerevisiae* strain *ScCop6* and time course analysis

pHis8Cop6 was used as a template for the amplification of the gene to be cloned in the p426GPD episomal vector for the expression of *Cop6* in *S. cerevisiae*. The gene was amplified with primers flanked with the restriction sites for *EcoRI* and *SpeI* and was mutated in positions 497 and 558 to eliminate the native *EcoRI* sites. The fragment was then cloned in the previously digested vector and the complete construct was used to transform the *S. cerevisiae* wild type strain to obtain *ScCop6*. Positive colonies were selected on plates containing minimal medium without uracil and one positive colony was isolated and used for the time courses.

Similarly to the time course performed for *EcCop6*, eight 100-ml flasks with 10 ml medium and 500 μl of dodecane and hexadecane solution were incubated at 30°C at 200 rpm. The four time points were chosen 24, 48, 72 and 96 hours from the inoculation in the fresh medium and the initial OD_600_ of the fresh cultures was 0.05 for all the experiments.

### Isolation and characterization of *X. dendrorhous* mutants

The plasmids pCrtE-PSS, pCrtYB-PSS and pPR-PSS [12]were used as backbone for the creation of the new plasmids pCrtE-Cop6, pCrtYB-Cop6 and pPR-Cop6, respectively. *Cop6* was amplified from the mutated gene used for the expression in *S. cerevisiae* and was flanked by the restriction sites for *NheI* and *SalI*. The fragment was then cloned in the digested vectors and used for the transformation of *X. dendrorhous*. The positive colonies were isolated for the ability to grow on selective medium with geneticin. From the transformations with pCrtE-Cop6, pCrtYB-Cop6 and pPR-Cop6, the three new mutant strains *ΔE-Cop6*, *ΔYB-Cop6* and *XdCop6* were obtained, respectively (Figure 2). The three strains were grown at 21°C at 200 rpm for the analysis of production of α-cuprenene in time following the same conditions used for the time course for *ScCop6*.

### GC-MS analysis

The dodecane solutions isolated from the different cultures at different time points were diluted 1:10 in ethyl acetate and run on GCMS to reveal and quantify the α-cuprenene production. A Shimadzu GCMS-QP5000 provided with a ZB-1 ms dimethylpolysiloxane column (Phenomenex 0.25 mm inner diameter, 0.25 μm thickness, 15 m length) was used for the analysis. Two microliters of diluted dodecane were injected splitless and analyzed in total ion scan using helium as carrier gas. The GCMS program consisted of an oven initial temperature of 50°C with an increment of 5°C/min up to 105°C and then up to 200°C with an increase of 30°C/min. The quantitation of the α-cuprenene was based on the hexadecane peak which had a known concentration.

### Microscopy analysis and cell counting

Aliquots of *ScCop6* and *XdCop6* cultures were taken after 96 hours of growth, diluted to OD_600_ 0.15, approximately, and were then transferred to a Bürker counting chamber (Bright line, Labor Optik). The number of cells counted in a surface of 0.0025 mm^2^ was multiplied by 10^4^ and divided by the OD6_600_ values of the cultures to obtain the number of cells per OD_600_ unit.

A Leica DM 6000B microscope provided with a 40x magnification objective and the LAS AF program was used for the visualization and to measure the size of the cells with the 10 μm bar provided by the LAS AF program.

## Competing interest

The authors declare that they have no competing interests.

## Authors’ contributions

EM conceived, designed and performed all the experiments. RS contributed to the microscopy analyses. WQ and OK supervised the research. All the authors read and approved the manuscript.
